# Construction of an Antibiotic-Free Vector and its Application in the Metabolic Engineering of *Escherichia Coli* for Polyhydroxybutyrate Production

**DOI:** 10.3389/fbioe.2022.837944

**Published:** 2022-06-03

**Authors:** Ying-Cheng Liao, Boonyawee Saengsawang, Jun-Wei Chen, Xiao-Zhen Zhuo, Si-Yu Li

**Affiliations:** ^1^ Department of Chemical Engineering, National Chung Hsing University, Taichung, Taiwan; ^2^ Innovation and Development Center of Sustainable Agriculture, National Chung Hsing University, Taichung, Taiwan

**Keywords:** antibiotic-free vector, hok/sok, Alp7A, inducer-free fermentation, polyhydroxybutyrate

## Abstract

An antibiotic- and inducer-free culture condition was proposed for polyhydroxybutyrate (PHB) production in recombinant *Escherichia coli*. First, antibiotic-free vectors were constructed by installing the plasmid maintenance system, *alp7, hok/sok*, and the *hok/sok* and *alp7* combination into the pUC19 vector. The plasmid stability test showed that pVEC02, the pUC19 vector containing the *hok/sok* system, was the most effective in achieving antibiotic-free cultivation in the *E. coli* B strain but not in the K strain. Second, the putative *phaCAB* operon derived from *Caldimonas manganoxidans* was inserted into pVEC02 to yield pPHB01 for PHB production in *E. coli* BL21 (DE3). The putative *phaCAB* operon was first shown function properly for PHB production and thus, inducer-free conditions were achieved. However, the maintenance of pPHB01 in *E. coli* requires antibiotics supplementation. Finally, an efficient *E. coli* ρ factor-independent terminator, thrLABC (ECK120033737), was inserted between the *phaCAB* operon and the *hok/sok* system to avoid possible transcriptional carry-over. The newly constructed plasmid pPHB01-1 facilitates an antibiotic- and inducer-free culture condition and induces the production of PHB with a concentration of 3.0 on0.2 g/L, yield of 0.26 /L0.07 g/g-glucose, and content of 44 /g3%. The PHB production using *E. coli* BL21 (DE3)/pPHB01-1 has been shown to last 84 and 96 h in the liquid and solid cultures.

## Introduction

Plasmid-based genetic manipulation is a common approach in metabolic engineering or whole-cell bio-catalysis. One advantage of employing plasmid-based genetic manipulation is the permit of *in vitro* genetic manuscription. The maintenance of recombinant plasmids in bacterial cells usually depends on the use of selective conditions, such as antibiotics supplementations. However, the scaling up of antibiotics use is expensive.

Strategies for the maintenance of plasmids during cell growth under antibiotic-free conditions have been extensively studied (Zielenkiewicz and Cegłowski, 2001; Kroll et al., 2010; Akiyama et al., 2011; Laguna et al., 2015; Singha et al., 2017). Among such strategies, post-segregational killing (PSK) and active partition systems have been proven to increase plasmid stability (Nordström and Austin, 1989; Sayeed et al., 2000). The *hok/sok* (formerly *parB*) locus, following the PSK mechanism, is a toxin-antidote system that simultaneously produces a toxin (Hok) and a short-lived antidote (Sok), such that in the event of plasmid loss, the cell will be killed by the long-lived toxin (Wu and Wood, 1994; Pecota et al., 2003; Friehs, 2004; Danino et al., 2015). In other words, only the cell that harbors the *hok/sok* locus can propagate because of Sok production.

The *alp7* system, found in *Bacillus subtilis*, is an active partition system consisting of two acting proteins (Alp7A and Alp7R; actin-like proteins) and a centromere-like cis-acting site (alp7C). Alp7A protein is an ATPase which interact with the Alp7R protein. The Alp7R protein recognizes and binds to the *alp7C* site, and the formation of the Alp7CR complex with the help of Alp7A pushes plasmids to the cellular poles during plasmid replication, ensuring equal segregation during cell division (Friehs, 2004; Derman et al., 2009; Derman et al., 2012; Million-Weaver et al., 2014).

Wu and Wood showed that *Escherichia coli* BK6 harboring a *hok/sok-*containing system maintained the plasmid for over 300 h without antibiotics (reaching a 90% proportion of antibiotic-resistant cells) (Wu and Wood, 1994). A recombinant plasmid has been shown to be maintained in *E. coli* Nissle 1917 over 72 h with the help of a *hok*/*sok* and *alp7* combination so that the recombinant *E. coli* Nissle 1917 can serve as a programmable probiotic for cancer detection (Danino et al., 2015). A recombinant plasmid harboring the *alp7* system can be maintained in *B. subtilis* over 14 generations without the supplementation of antibiotics (Derman et al., 2009; Derman et al., 2012). A *trpA*-containing plasmid can be maintained in *E. coli* M72 (K-strain) over 100 generations by integrating the *hok/sok* locus in the plasmid backbone (Kim and Kang, 1996).

Poly-3-hydroxybutyrate (PHB) has attracted much attention owing its biodegradability and biocompatibility, particularly because of the increase in global environmental concerns in recent years. The common method for PHB biosynthesis from acetyl-CoA in microorganisms comprises three steps. First, two molecules of acetyl-CoA are condensed by β-ketothiolase, encoded by *phaA* gene, to form acetoacetyl-CoA. Second, acetoacetyl-CoA reductase (encoded by *phaB*) converts acetoacetyl-CoA to 3-hydroxybutyryl-CoA using NADPH. Finally, the enzyme PHA synthase (encoded by *phaC*) polymerizes 3-hydroxybutyryl-CoA monomers to PHB, liberating CoA (Bernd, 2003; Stubbe et al., 2005; Peña et al., 2014; Boontip et al., 2021). In our previous study, we expressed the PHB biosynthesis genes, *phaCAB*, from the thermophilic *Caldimonas manganoxidans* in *E. coli* and achieved PHB production with a concentration of 16.8 ± 0.6 g/L, content of 74%, and yield of 0.28 g/g glucose (Lin et al., 2017). The expression of the three genes was achieved by inserting *phaA* and *phaB* into the commercial vector, pCDF-Duet1, and inserting *phaC* into a vector, T-BAD, which is ampicillin resistant.

In this study, we constructed three pUC19-based vectors, pVEC01, pVEC02, and pVEC03, which contain *alp7, hok/sok*, and the *alp7-hok/sok* combination, respectively. The stability of pVEC01-03 without the ampicillin supplementation was tested in various *E. coli* strains. The most stable vector among the three was chosen for application in PHB production. The putative *phaCAB* operon of *C. manganoxidans* was first amplified from the *C. manganoxidans* genome, where the amplicon (3,919 bp in total) contained the *phaCAB* gene cluster and a 194-bp fragment upstream of *phaC*. In this manner, the expression of *phaCAB* genes will be under the control of the promoter and ribosome binding site (RBS) from *C. manganoxidans*. This is practically important because any possible inducer, such as IPTG, can be avoided for the economics purpose, where IPTG was calculated to cost 10% of total cost of recombinant β-glucosidase production (Ferreira et al., 2018). While the *phaCAB* operon has been shown to be functionalized in *E. coli* in this study, the function of the recombinant plasmid containing the putative *phaCAB* operon in *E. coli* can only be shown with ampicillin supplementation in the first trial. The latter part of the study proposes a solution to take advantage of the *hok/sok* for antibiotic-free PHB production.

## Materials and Methods

### Bacteria Strains and Plasmids

All strains and plasmids used in this study are listed in Table 1.

**TABLE 1:** List of bacterial strains and plasmids used in this study.

Name	Descriptions	References
Bacterial strains		
*E. coli* DH5α	F^−^ endA1 glnV44 thi-1 recA1 relA1 gyrA96 deoR nupG purB20 φ80dlacZΔM15 Δ(lacZYA-argF)U169, hsdR17 (rK^–^mK^+^), λ^–^	Lab stock
*E. coli* BL21 (DE3)	F^−^, *dcm*, *ompT*, *gal*, *lon*, *hsd*S_B_(rB^−^, mB^−^), λ(DE3 [*lac*I, *lac*UV5-T7 gene 1, *ind*1, *sam*7,*nin*5])	Lab stock
*E. coli* MG1655	F^−^ lambda^−^ *ilvG*- *rfb*-50 *rph*-1	Lab stock
*E. coli* MZLF	*E. coli* BL21 (DE3) Δ*zwf*, Δ*ldhA*, Δ*frd*	Yang et al. (2016)
*C. manganoxidans*	JCM 10698 (BCRC 17858)	Lab stock
Plasmids		
pUC19	Commonly used cloning vector that conveys Tet and Amp resistance.	Lab stock
pTKW106alp7A	Recombinant plasmid carries *alp7CAR* gene and *hok/sok* gene	Danino et al. (2015)
pVEC01	Recombinant plasmid carries *alp7CAR* gene (derived from *B. subtilis* pLS20) to maintain the plasmid stability.	This study
pVEC02	Recombinant plasmid carries *hok/sok* gene (derived from *E. coli* R1 plasmid) to maintain the plasmid stability.	This study
pVEC03	Recombinant plasmid carries *alp7CAR* gene and *hok/sok* gene to maintain the plasmid stability.	This study
pPHB01	pVEC02-based plasmid carries *hok/sok* gene and *phaCAB* operon (derived from *C. manganoxidans*) under the control of the native promoter of *phaCAB* operon from *C. manganoxidans*.	This study
pPHB01-1	pPHB01 derived recombinant plasmid where a terminator was inserted between *hok/sok* gene and *phaCAB* operon	This study

### DNA Manipulation and Transformation

**FIGURE 1:**
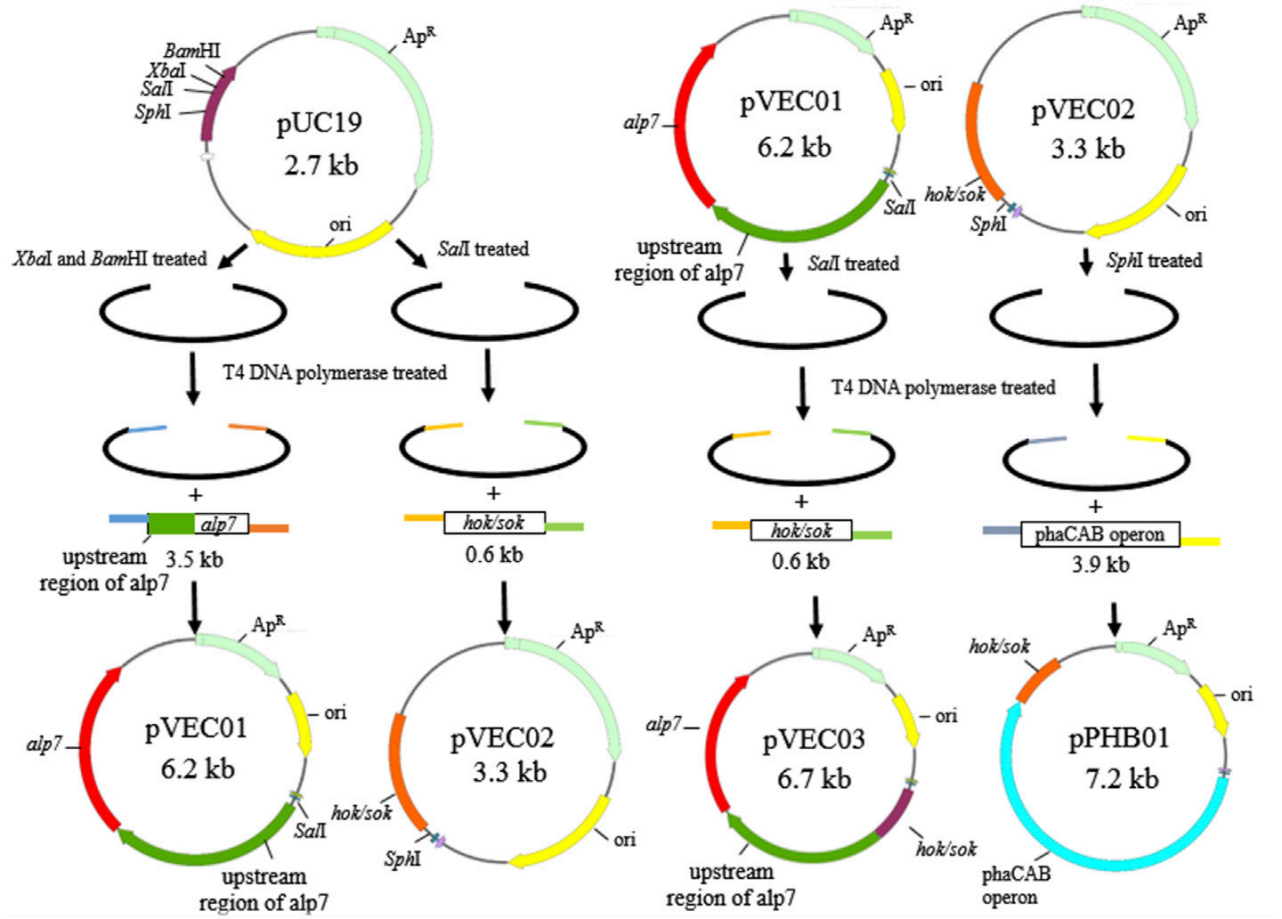
Schematic of the construction of pVEC01, pVEC02, pVEC03, and pPHB01.

Plasmids were constructed by sequence- and ligation-independent cloning (Li and Elledge, 2007; Jeong et al., 2012; Islam et al., 2017) and schematic diagrams of plasmid construction are shown in Figure 1 The primers used in this study are listed in Table 2. To construct the recombinant plasmid pVEC01, *alp7* was amplified from pTKW106alp7 (Addgene, Watertown, United States) by polymerase chain reaction (PCR), and pUC19 was digested with *Xba*I and *Bam*HI. To construct the recombinant plasmids pVEC02 and pVEC03, the *hok/sok* locus was amplified from pTKW106alp7 by PCR, and the vectors pUC19 and pVEC01 were digested with *Sal*I. To construct the recombinant plasmid pPHB01, the *phaCAB* operon was amplified from the genomic DNA of *C. manganoxidans* and pVEC02 was digested with *Sph*I. After the preparation of inserts and vectors, the DNA fragments were purified using the Gene-Spin™ 1-4-3 DNA Extraction Kit (PROTECH, Taipei, Taiwan). The insert and corresponding vector were mixed at an appropriate ratio and incubated at 37°C for 1 min with T4 DNA polymerase to generate 5′-overhangs. Thereafter, the reaction mixture was placed on ice for 20 min for single-strand annealing and was introduced into competent *E. coli* DH5α cells for transformation.

**TABLE 2:** Primer sequence in this study.

Primer	Sequence^a^
SLIC-F-alp7-i	TGC​CTG​CAG​GTC​GAC​TCT​AGa​cca​cct​agg​tca​tta​gcc​t
SLIC-R-alp7-i	GAG​CTC​GGT​ACC​CGG​GGA​TCt​cag​ggc​gtc​tgt​gtt​gca​a
SLIC-F-parB-01	CTT​GCA​TGC​CTG​CAG​GTC​GAa​aca​aac​tcc​ggg​agg​cag​c
SLIC-R-parB-01	CCT​AGG​TGG​TCT​AGA​GTC​GAa​caa​cat​cag​caa​gga​gaa​a
SLIC-F-phaCAB1-01	CCA​TGA​TTA​CGC​CAA​GCT​TGg​gta​cat​gga​gca​gat​gcg​c
SLIC-R-phaCAB1-01	AGT​TTG​TTT​CGA​CCT​GCA​GGc​gag​ttg​atc​gcc​aac​gaa​g
gko-F-CAB200-01	ATGAAGCGAACCACCTCC
gko-R-CAB200-01	CAA​GCT​TGG​CGT​AAT​CAT​G
In/del-F- pPHB01-1	CCC​GCA​CCT​GAC​AGT​GCG​GGC​TTT​TTT​TTT​CGA​CCA​AAG​Gag​tgc​cgc​tct​tcg​ttg​gcg​atc​aac​tcg
In/del-R- pPHB01-1	CTT​TTT​Tct​gtg​ttt​ccA​TTG​TTA​GAC​GAG​AGT​GTG​CTC​AGT​TGT​CAA​Gcc​gtc​cgc​cgg​gac​ggc​cgc​atg​tcc​tgg​ctt​ac
CK-F-pPHB01-1 (in-del)	GAA​GAA​ATC​GCT​TCG​ATC​G
CK-R-pPHB01-1 (in-del)	AAA​CCA​CCT​TCA​CGT​CAT​G

a Uppercase letters indicate regions of homology with vector; lowercase letters indicate the primer for insert DNA.

To construct the recombinant plasmid pPHB01-1, the Q5^®^ site-directed mutagenesis kit (NEB, Ipswich, United States) was used to insert a 57-bp *E. coli* ρ factor-independent terminator, thrLABC (ECK120033737) between the *phaCAB* operon and the *hok/sok* locus in pPHB01. The primers used for constructing pPHB01-1 were In/del-F- pPHB01-1 and In/del-R-pPHB01-1 (Table 2). After PCR, the amplified DNA was treated with a Kinase-Ligase-DpnI enzyme mix at room temperature for 5 min for circularization and template removal. Mutant selection was enriched by simultaneous ligation and *Dpn*I treatment. Next, 5 μL of the KLD mix was directly introduced into competent *E. coli* DH5α cells. Colony PCR was used for pPHB01-1 screening with the primers CK-F-pPHB01-1 (in-del) and CK-R-pPHB01-1 (in-del) (Table 2). Finally, pPHB01-1 was confirmed by DNA sequencing. All recombinant plasmids constructed in this study were verified by colony PCR, restriction enzyme digestion, and DNA sequencing.

### Plasmid Stability Test

*Escherichia coli* strains DH5α, MG1655, BL21 (DE3), and MZLF (Yang et al., 2016) were used as hosts to test plasmid stability. Cells for the plasmid stability test were grown from -80°C stocks by streaking on an LB plate with ampicillin. After 16–24 h, a single colony was picked and used to inoculate LB containing antibiotics for 12 h. Then, 10 μL of the cell suspensions was inoculated into 3 ml LB without antibiotics to start the stability experiment. The inoculum was diluted and plated on LB plates supplemented with or without antibiotics.

After 12 h, 10 μL of the cell suspensions was transferred to a new test tube. Every 12 h, 10 μL of the sample was transferred to a new flask, and the cells were diluted and plated on LB plates supplemented with or without antibiotics. Plasmid stability (segregation) was estimated by the equation:

$$\text{Ampicillin}-\text{resistant cells }\left(\%\right)\;=\frac{\text{colonies on}\;LB+\text{ampicillin}}{\text{colonies on LB}}\times 100\%.$$

### Characterization of the Putative Promoter of the *phCAB* Operon From *C. Manganoxidans*

The putative promoter of the *phaCAB* operon was predicted by BPROM (http://www.softberry.com/berry.phtml) and putative ribosome binding sites (RBSs) were reported in a previous study (Lin et al., 2017).

### Culture Conditions for PHB Production

The strains used for shake flask experiments were grown aerobically at 200 rpm and 37°C in fresh 25-ml LB medium supplemented with 20 g/L glucose, 0.6 g/L MgSO_4_ 7H_2_O, 0.07 g/L CaCl_2_ 2H_2_O, 0.04 g/L KH_2_PO_4_, 0.04 g/L K_2_HPO_4_, 0.4 g/L NaHCO_3_ and 100 μg/ml ampicillin. In the shake flask experiments, the initial OD_600_ was adjusted to 0.05. Samples were taken and stored every 12 h until 48 h for the subsequent analysis.

### Analytical Methods

Cell density was monitored by measuring the optical density at 600 nm (GENESYS 10S, Thermo Scientific, United States). For the measurement of biomass concentration, 3 ml of the cultured cells were centrifuged for 1 min at 6,791 rcf. The cells were frozen at −20°C and lyophilized. After freeze-drying, the weight of the sample was recorded to calculate the biomass concentration. The PHB produced by the recombinant *E. coli* cells was quantified by gas chromatography (GC), and the samples were treated as described before (Hsiao et al., 2016). Briefly, an appropriate amount of biomass was transferred to a clean spiral test tube and 1 ml of chloroform, 0.85 ml of methanol, and 0.15 ml of sulfuric acid were added. The tube was incubated in a water bath for 140 min at 80°C and cooled down to room temperature. Then 1 ml of DI H_2_O was mixed well by vortexing. After standing and layering, the organic phase was removed and filtered through a 0.2-μm PVDF filter and then analyzed by GC. The temperatures of the injector and detector were 230 and 275°C, respectively. The temperature of the column was set at 100°C and increased to 200°C at a rate of 10°C/min and maintained at 200°C for 2 min. The nitrogen was used as the carrier gas at 3 ml/min. The split mode with the split ratio of 1:10 was used (Chen S.-K. et al., 2013).

The metabolites were determined using a Thermo Scientific™ Dionex™ Ulitmate 3000 LC system equipped with an ORH-801 column. Sulfuric acid (5 mM) was used as the mobile phase, and the flow rate was maintained at 0.6 ml/min. Samples for quantification were collected from the culture media after the removal of the cells by centrifugation for 1 min at 6,791 rcf. The supernatant was passed through a 0.2-μm PVDF filter, and the sample injection (10 μL) was performed using an auto-sampler.

### Sodium Dodecyl Sulphate-Polyacrylamide Gel Electrophoresis

The bacterial culture solutions were centrifuged at 4°C and 9,072 × g for 10 min and resuspended in lysis buffer (50 mM Tris, 0.1 M NaCl, 1 mM EDTA, pH 7.8) to obtain a final OD of 20. The concentrated bacterial solutions were subjected to ultrasonication (10 s break and 5 s intermittent, total 60 cycle) (QSonica Q700, United States). The cell lysates were then diluted with 4x Laemmli sample buffer (Bio-Rad Laboratories, Inc., United States) and heated in a dry batch for 10 min. Samples (10 µL) were loaded into the wells for SDS-PAGE analysis (12% acrylamide).

### Nile Red Stain Assay

To confirm *in vivo* PHB production, the recombinant *E. coli* BL21 (DE3)/pPHB01 was grown on LB plates containing 10 g/L glucose and 0.8 mg/ml nile red. After 24 h of cultivation at 37°C, the plates were exposed to monochromatic light at 470 nm for observation.

## Results

### Stability of pVEC01, pVEC02, and pVEC03 in *E. coli*

**FIGURE 2:**
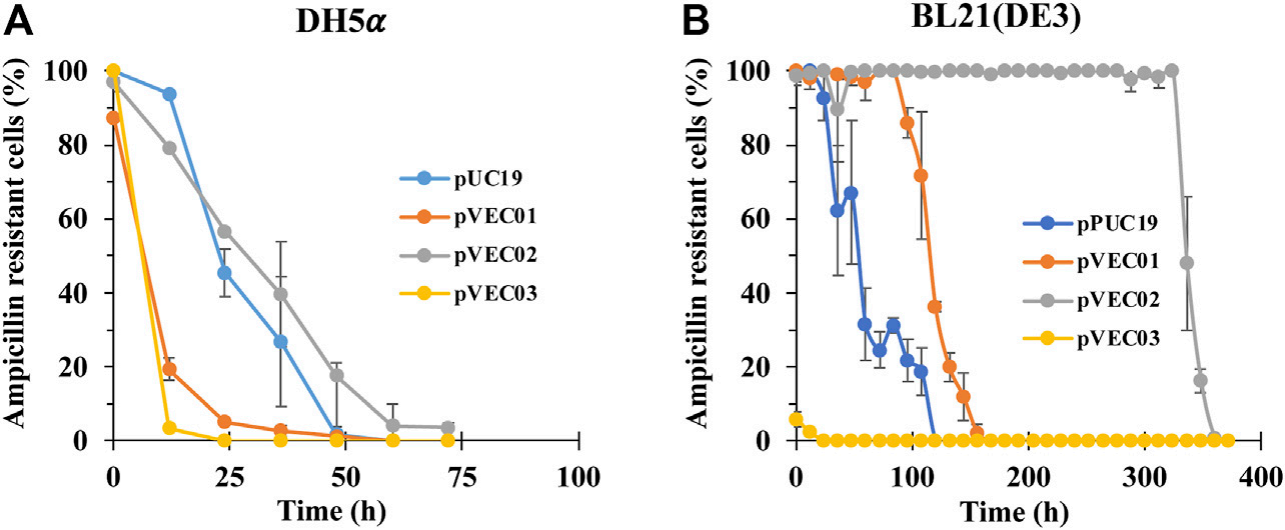
The plasmid stability test of pUC19, pVEC01, pVEC02, and pVEC03 in *E. coli* strains **(A)** DH5α and **(B)** BL21 (DE3). Errors represent standard deviation with *n* = 3.

The recombinant plasmids pVEC01, pVEC02, and pVEC03 were tested for plasmid stability in different *E. coli* hosts. Compared to the parental plasmid pUC19, pVEC02 showed good stability in *E. coli* DH5α *E. coli* BL21 (DE3) and with time required to reach 50% ampicillin-resistant cells of 26 and 336 h, respectively (while pUC19 had times of 25 and 54 h, respectively, Figures 2A,B). The *hok/sok* system increased the segregational stability in BL21 (DE3) by 6 fold, but not in *E. coli* K strain. In fact, pVEC01, pVEC02, and pVEC03 were not stable in *E. coli* K strains DH5α. pVEC01 was slightly more stable than pUC19 in BL21 (DE3) cells and pVEC03 showed instability. Because of the stability of pVEC02 in this test, it was chosen as the new vector for the subsequent induction of PHB production.

### PHB Production by *E. Coli* BL21(DE3)/pPHB01 in Shake Flasks

**FIGURE 3:**
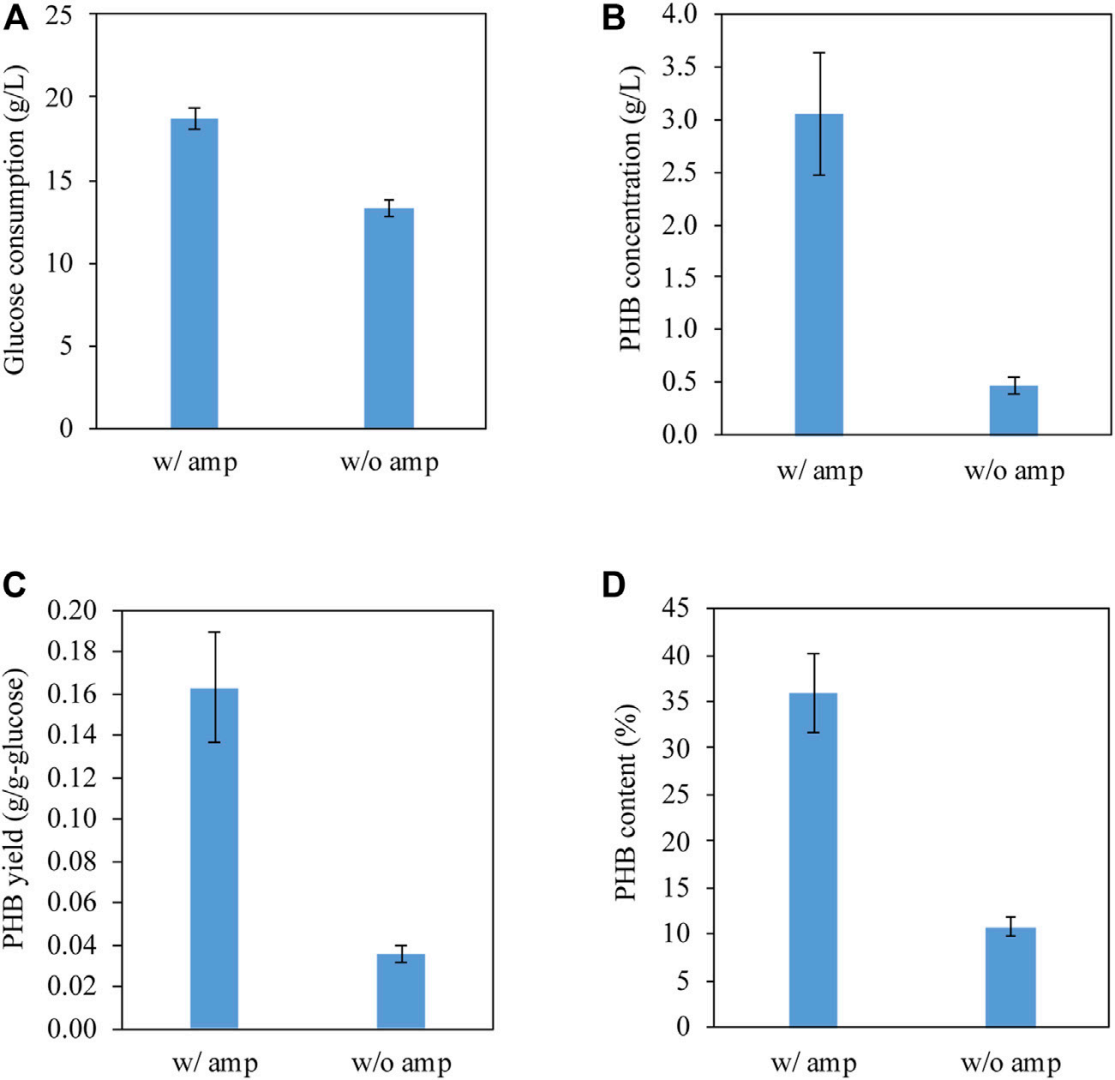
**(A)** Glucose consumption, **(B)** PHB concentration, **(C)** PHB yield, and **(D)** PHB content of *E. coli* BL21 (DE3)/pPHB01 with and without ampicillin supplementation. The initial glucose concentration for conditions of with and without amp were 19.6 BL0.4 and 18.7 .40.5 g/L, respectively. Errors represent standard deviation with *n* = 3.

We tested PHB production by conducting shake flask experiments at 37°C under the control of the native promoter of *phaCAB* operon from *C. manganoxidans*. Figure 3A shows that *E. coli* BL21 (DE3)/pPHB01 consumed 18 DE1 and 13 a1 g/L glucose with and without ampicillin, respectively. In the presence of ampicillin, *E. coli* BL21 (DE3) harboring pPHB01 was able to effectively produce PHB and achieved a PHB concentration of 3.1 DE0.6 g/L (Figure 3B), yield of 0.16 , 0.03 g/g-glucose, and content of 36 /g4% (Figure 3D). In contrast, in the absence of ampicillin, *E. coli* BL21 (DE3) harboring pPHB01 produced PHB with a decreased concentration of 0.5 w0.1 g/L, as shown in Figures 3B–D. The effective PHB production in *E. coli* BL21 (DE3)/pPHB01 justifies the high glucose consumption in the presence of ampicillin.

**FIGURE 4:**
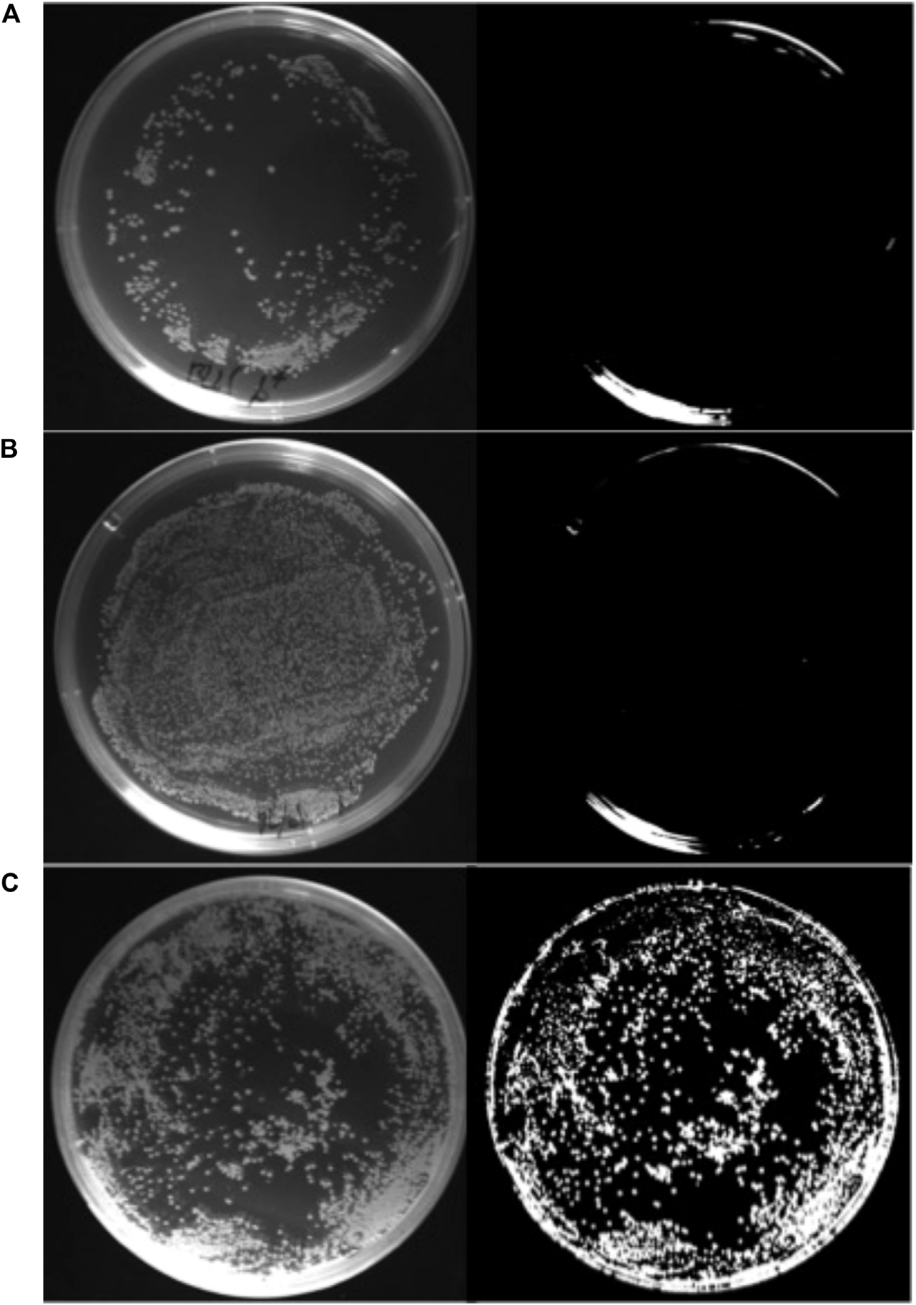
Nile red stain assay of **(A)** *E. coli* BL21 (DE3), **(B)** *E. coli* BL21 (DE3)/pPHB01 **(C)** *E. coli* BL21 (DE3)/pPHB01-1. The LB agar plate contained 10 g/L glucose and 0.8 mg/ml nile red without ampicillin. The plate on the left and right in each panel was taken under white light and monochromatic light at 470 nm, respectively.

The capability of *E. coli* BL21 (DE3)/pPHB01-1 to produce PHB without the inducer and antibiotics was confirmed by the Nile red assay, as shown in Figure 4.

### Characterization of the *phaCAB* Operon From *C. manganoxidans*

**FIGURE 5:**
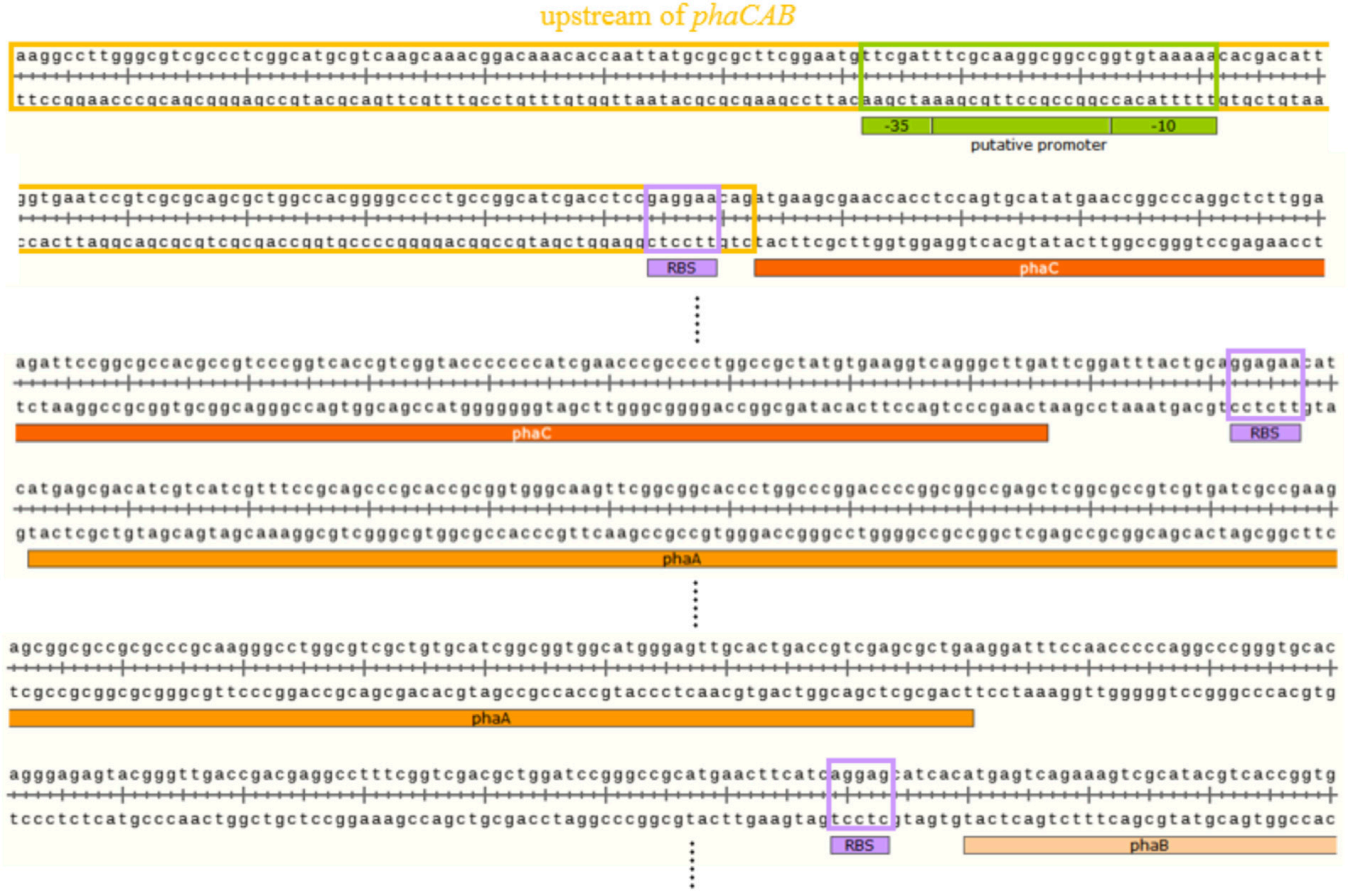
The putative promoter of *phaCAB* operon and the putative ribosome binding sites (RBSs) of *phaCAB* genes.

The putative promoter of the *phaCAB* operon was predicted by BPROM, and the sequences of the putative promoter, -35 box and -10 box sequences were TTC​GAT​TTC​GCA​AGG​CGG​CCG​GTG​TAA​AAA, TTCGAT, and GTGTAAAAA, respectively (Figure 5). Furthermore, RBSs of the *phaCAB* operon were labeled according to a previous study (Figure 5) (Lin et al., 2017).

### Isolation of the *phaCAB* Operon and *Hok/Sok* Genetic Units by Inserting a Terminator

**FIGURE 6:**
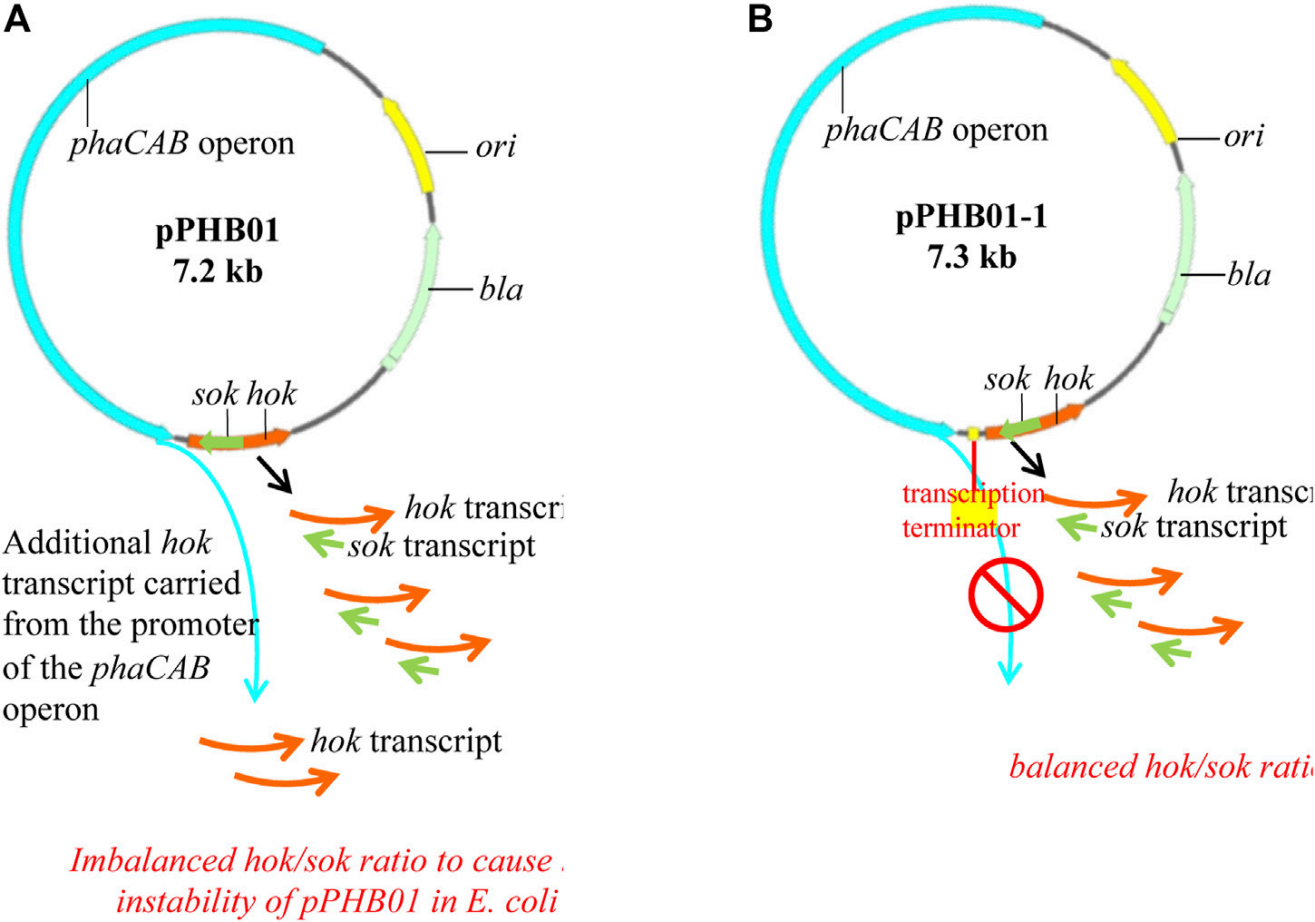
Maps of **(A)** pPHB01 and **(B)** pPHB01-1, where pPHB01-1 was constructed by inserting a 57-bp *E. coli* ρ factor-independent terminator (thrLABC, ECK120033737) into the pPHB01 to isolate the *phaCAB* operon and the *hok/sok* system.

While pVEC02 (*hok/sok*) was shown to be a stable vector in *E. coli* without ampicillin supplementation, the low PHB production by *E. coli* BL21 (DE3)/pPHB01 in the absence of ampicillin indicates the instability of pPHB01 in *E. coli* BL21 (DE3). It is speculated that the insertion of the *phaCAB* operon in pVEC02 disrupted the balance of hok/sok transcripts. In other words, the promoter of the *phaCAB* operon may carry an additional transcript of *hok* and make pPHB01 toxic to *E. coli* (Figure 6). To verify this speculation, an efficient *E. coli* ρ factor-independent terminator, thrLABC (ECK120033737) (Chen Y.-J. et al., 2013), was inserted between the *phaCAB* operon and the *hok/sok* locus to isolate the two genetic units (Figure 6). The newly constructed plasmid was named pPHB01-1.

**FIGURE 7:**
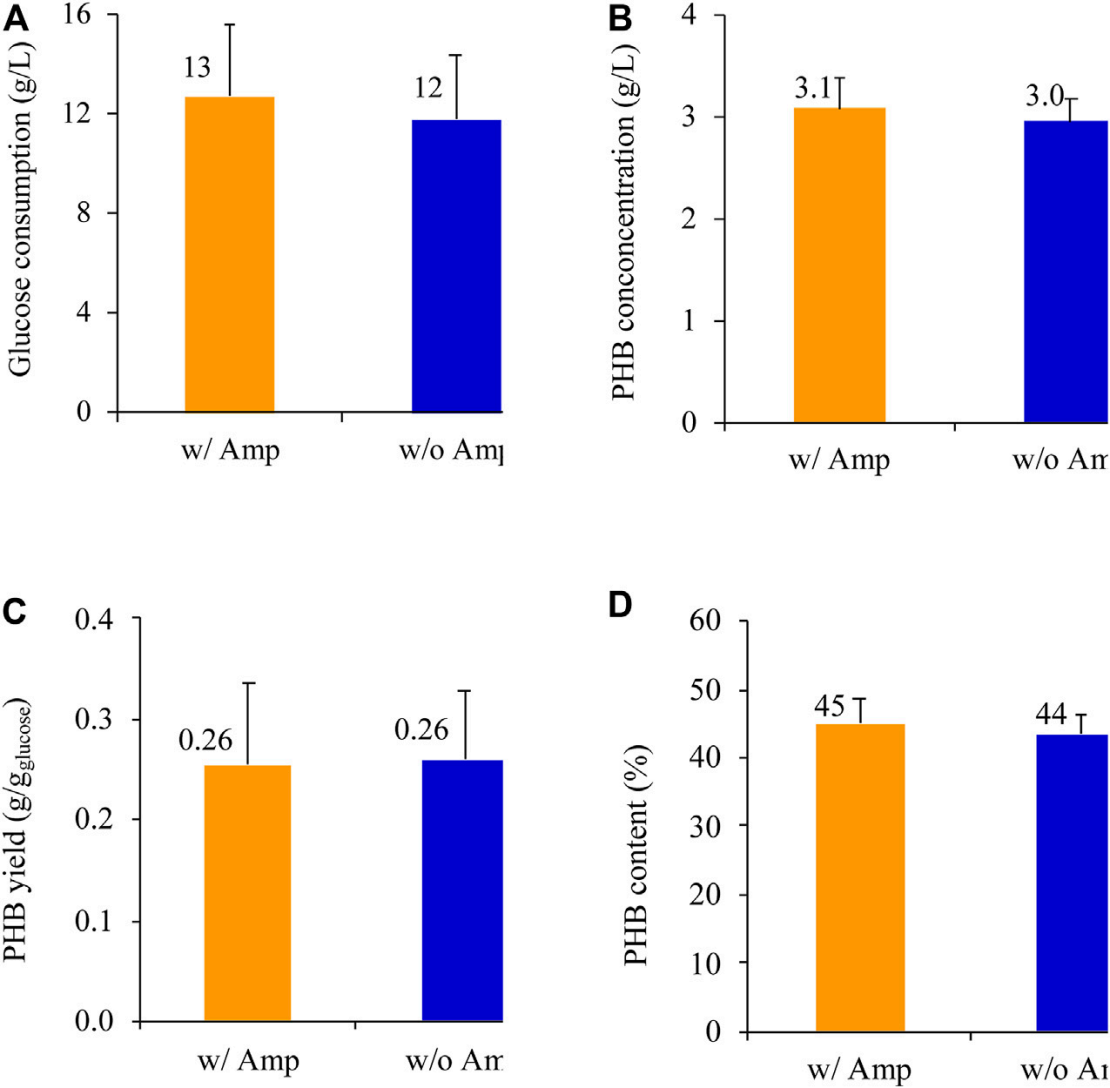
**(A)** Glucose consumption, **(B)** PHB conconcentration, **(C)** PHB yield, and **(D)** PHB content of *E. coli* BL21 (DE3)/pPHB01-1 with out and with 100 μg/ml ampicillin. The initial glucose concentration for conditions of with and without amp were 13 am3 and 12 a3 g/L, respectively. Errors represent standard deviation with *n* = 3.

*E. coli* BL21 (DE3) harboring the newly constructed plasmid pPHB01-1 in was tested for the PHB production with and without ampicillin supplementation (Figure 7). In the presence of ampicillin, *E. coli* BL21 (DE3)/pPHB01-1 was able to produce PHB effectively. All the fed glucose of 13 DE3 g/L was completely consumed in 48 h (Figure 7A), and the PHB concentration, yield, and content were 3.1 , 0.3 g/L, 0.26 /L0.08 g/g-glucose, and 45 /g4%, respectively (Figures 7B–D). In the absence of ampicillin supplementation, *E. coli* BL21 (DE3)/pPHB01-1 was still able to provide a comparable PHB performance, where the PHB concentration, yield, and content was 3.0 PH0.2 g/L, 0.26 /L0.07 g/g-glucose, and 44 /g3%, respectively (Figures 7B–D).

**FIGURE 8:**
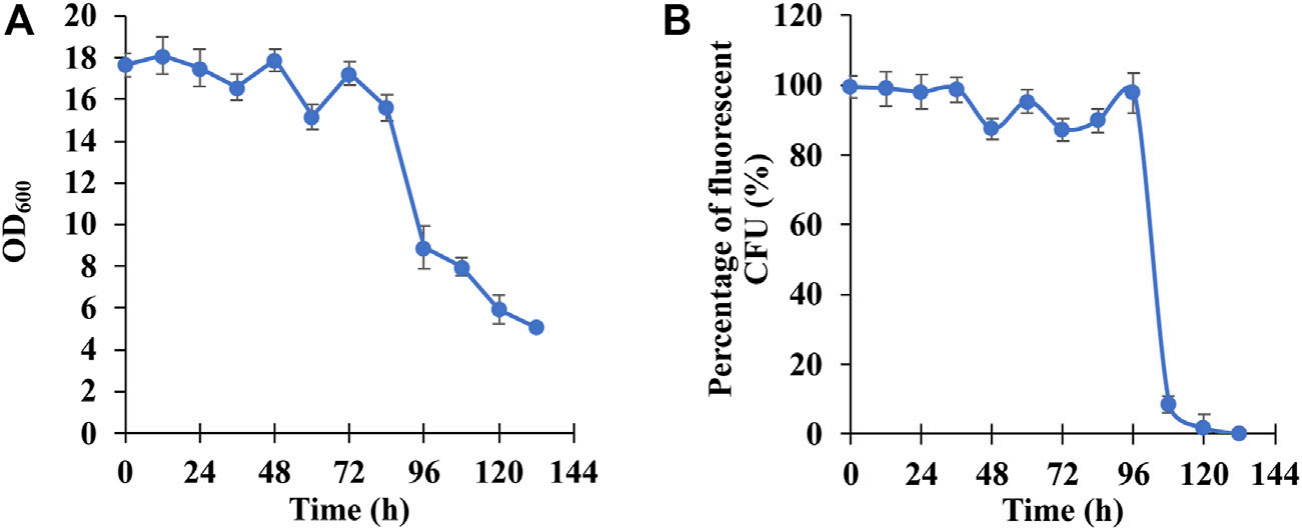
**(A)** OD_600_ and **(B)** percentage of fluorescent colony forming unit (CFU) of *E. coli* BL21 (DE3)/pPHB01-1 for the long-term PHB production. Errors represent standard deviation with *n* = 3.

To characterize the long-term stability of PHB production in *E. coli* BL21 (DE3)/pPHB01-1, *E. coli* BL21 (DE3)/pPHB01-1 was first cultivated in the liquid culture without the ampicillin (the medium composition was described in 2.5) and transferred to a fresh medium every 12 h. During the transfer, the grown-culture solution was spread on the agar plate without the ampicillin but 0.8 mg/ml nile red. After the formation of CFU, the total CFU as well as the fluorescent CFU were counted to quantify the percentage of CFU that can produce PHB. It can be seen in Figure 8A that the OD_600_, which is positively correlated to the PHB production, can be maintained in the range of 15 and 18 in the first 84 h and gradually dropped to 5.1 a0.1 at 132 h. Figure 8B shows that the *E. coli* BL21 (DE3)/pPHB01-1 maintained its ability to produce PHB in the first 96 h and less than 20% of cell population can produce PHB afterwards.

### SDS-PAGE of *E. Coli* BL21(DE3)/pPHB01 and *E. Coli* BL21(DE3)/pPHB01-1

**FIGURE 9:**
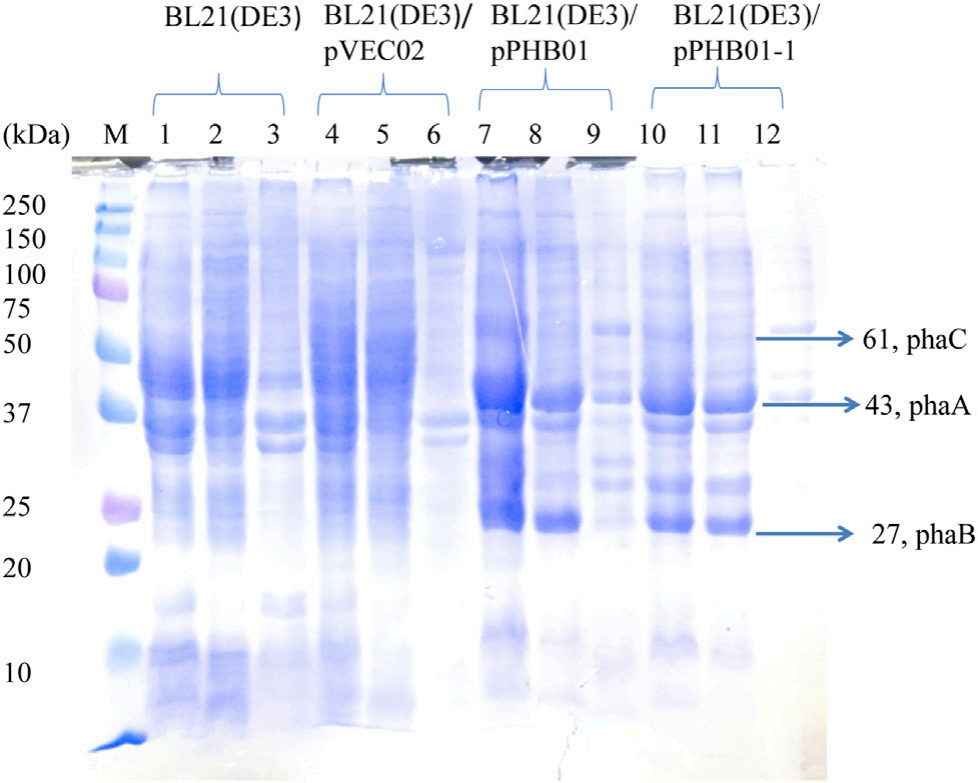
SDS-PAGE of *E. coli* BL21 (DE3)/pPHB01 and BL21 (DE3)/pPHB01-1. M: marker; lane 1- lane 3: BL21 (DE3); lane 4 - lane 6: BL21 (DE3)/pVEC02; lane 7- lane 9: BL21 (DE3)/pPHB01; lane 10 - lane 12: BL21 (DE3)/pPHB0-1. Lane 1, 4, 7, 10: whole cells; lane 2, 5, 8, 11: soluble protein; lane 3, 6, 9, 12: insoluble fractions.

SDS-PAGE was used to confirm the expression of the *phaCAB* operon derived from *C. manganoxidans* in *E. coli*. It can be clearly seen in Figure 9 (lanes 7–12) that three overexpressed signals were found in both *E. coli* BL21 (DE3)/pPHB01 and *E. coli* BL21 (DE3)/pPHB01-1 samples, which correspond to PhaC (61 kDa), PhaA (43 kDa), and PhaB (27 kDa). PhaA and PhaB in *E. coli* BL21 (DE3)/pPHB01 and *E. coli* BL21 (DE3)/pPHB01-1 were mainly found in the soluble fractions (lanes 8 and 11 of Figure 9), while PhaC was mainly found in the insoluble fraction (lanes 9 and 12 of Figure 9).

## Discussion

Synthetic biology has gradually become the keystone of our current technology-based society (Tseng et al., 2018; Voigt, 2020; Pelletier et al., 2021). The management of synthetic biology relies on the characterization of genetic units and their interactions. Plasmid system is a general platform to carry genetic information and therefore plasmid instability becomes a concern to maintain the newly installed traits. In previous studies, *hok/sok* was found to be a potential system to increase the plasmid stability in *E. coli* strains (Gerdes et al., 1986; Schweder et al., 1992; Lee et al., 1994). Multiple copies of the *hok/sok* locus in the target plasmid can further improve the stability of the plasmid in *E. coli* (Gerdes et al., 1986; Lee et al., 1994; Kim and Kang, 1996; Pecota et al., 1997). However, it is common to see the expression of recombinant proteins cause the imbalance of the *hok/sok* system, where transcription and translation are simultaneously involved in the plasmid maintenance (Lee et al., 1994; Wu and Wood, 1994). Therefore, the combination of plasmid maintenance systems as a hybrid one was proposed to increase the plasmid stability and the time to reach 50% plasmid-bearing cells was extended to ∼ 72 h (Pecota et al., 2003).

In this study, a high-copy number backbone, pUC19, was chosen since it may benefit recombinant protein production and metabolic engineering. PSK and active partition systems are usually found in a low-copy number plasmid. It will be interesting to see how well PSK and active partition systems maintain the high-copy pUC19 in *E. coli*. This study clearly demonstrated that *alp7* system was incompatibility with the high-copy backbone. This may because the active partition of the plasmid consumes ATP during DNA replication (Baxter and Funnell, 2015). Energy consumption is critical for the function of *alp7*, and the installation of *alp7* in a high-copy number backbone may consume too much ATP, making *alp7*/pUC19 incompatible with *E. coli*. In contrast, the genetic unit *hok/sok* in pVEC02 was found to be an efficient antibiotic-free selection marker in *E. coli* B strains, including *E. coli* BL21 (DE3) and MZLF. The time to reach 50% ampicillin-resistant cells can go up to 360 h, which is competitive among literatures. Perplexedly, this study presented a result that the *hok/sok* system was not compatible with *E. coli* K strains, including MG1655 and DH5α. *E. coli* K and B strains bear 5 and 6 *hok/sok* loci in the chromosome, respectively (Schneider et al., 2002), and all of them are considered inactive yet may be induced by unknown signal (Pedersen and Gerdes, 1999). More study can be conducted in detail in the future to discriminate the compatibility of the *hok/sok* system among *E. coli* strains.

The application of the antibiotic-free vector pVEC02 was further investigated in metabolic engineering for PHB production by constructing the recombinant pPHB01. *E. coli* BL21 (DE3)/pPHB01 can effectively produce PHB; however, only in the presence of antibiotics. The ineffective function of the *hok/sok* system in pPHB01 demonstrates that the *hok/sok* system in the engineering perspective needs more detailed investigation. We focused on the interaction among genetic units to determine the antibiotic demand of pPHB01. It is speculated that the transcription activity of the *phaCAB* operon passes downstream of the *hok/sok* gene unit. The transcriptional carry-over increases *hok* transcription; therefore, the *hok/sok* balance is interrupted. This carry-over of the transcription activity can be especially severe when the downstream *hok* is a short transcript. To prevent transcriptional carry-over, an efficient *E. coli* ρ factor-independent terminator, thrLABC (ECK120033737) (Chen Y.-J. et al., 2013), was inserted between the *phaCAB* operon and the *hok/sok* gene unit (Figure 7). The isolation of the two genetic units was shown in this study to be effective in finding an application for pVEC02 in metabolic engineering. In addition, a putative promoter region of the *phaCAB* operon from *C. manganoxidans* was first shown properly while effectively function in *E. coli*. To our knowledge, this is the first study to demonstrate that the *phaCAB* operon from *C. manganoxidans* can be used for effective PHB production in *E. coli*. A previous study showed that the expression level of PhaC was important for the phaCAB-mediated pathway (Hiroe et al., 2012). As shown in Figure 9 that PhaC expression level was not strong, the PhaC expression level of the *phaCAB* operon in *E. coli* can be further optimized in the future. In summary, the inducer-free PHB production can be achieved by adopting heterologous promoter from *C. manganoxidans*. The antibiotic-free PHB production involves the interplay among bacteria chassis, antibiotic-free genetic markers, and the genetic operon for PHB production. This study presents a right combination to achieve the inducer- and antibiotic-free system for PHB production.

## Data Availability

The original contributions presented in the study are included in the article/Supplementary Material, further inquiries can be directed to the corresponding author.
